# Molecular mechanisms of resveratrol as chemo and radiosensitizer in cancer

**DOI:** 10.3389/fphar.2023.1287505

**Published:** 2023-11-10

**Authors:** Sandra Cotino-Nájera, Luis A. Herrera, Guadalupe Domínguez-Gómez, José Díaz-Chávez

**Affiliations:** ^1^ Laboratorio de Oncología Molecular, Departamento de Genética y Biología Molecular, Centro de Investigación y de Estudios Avanzados del Instituto Politécnico Nacional (CINVESTAV-IPN), Ciudad de México, Mexico; ^2^ Escuela de Medicina y Ciencias de la Salud-Tecnológico de Monterrey, México City, Mexico; ^3^ Subdirección de Investigación Clínica, Instituto Nacional de Cancerología (INCAN), Ciudad de México, Mexico; ^4^ Unidad de Investigación en Cáncer, Instituto de Investigaciones Biomédicas-Universidad Nacional Autónoma de México, Instituto Nacional de Cancerología, Ciudad de México, Mexico

**Keywords:** radioresistance, chemoresistance, resveratrol, cancer, molecular mechanisms

## Abstract

One of the primary diseases that cause death worldwide is cancer. Cancer cells can be intrinsically resistant or acquire resistance to therapies and drugs used for cancer treatment through multiple mechanisms of action that favor cell survival and proliferation, becoming one of the leading causes of treatment failure against cancer. A promising strategy to overcome chemoresistance and radioresistance is the co-administration of anticancer agents and natural compounds with anticancer properties, such as the polyphenolic compound resveratrol (RSV). RSV has been reported to be able to sensitize cancer cells to chemotherapeutic agents and radiotherapy, promoting cancer cell death. This review describes the reported molecular mechanisms by which RSV sensitizes tumor cells to radiotherapy and chemotherapy treatment.

## 1 Introduction

Cancer treatment and therapy have improved significantly in recent years, increasing patients’ survival and quality of life. However, cancer remains one of the main diseases with the highest mortality worldwide (Siegel et al., 2022). This poor prognosis in cancer patients is partly due to the adverse effects and complications that limit the patient’s survival and quality of life when using cancer treatments. But the main reason behind the failure of the most used therapies, such as chemotherapy, is cancer cells’ intrinsic or acquired resistance to the drugs. For example, cancer cells can evade the toxicity of drugs by developing resistance to them, which prevents the patient from getting better (Longley and Johnston, 2005; Holohan et al., 2013). On the other hand, radiotherapy is another treatment frequently used in cancer patients. However, the acquisition of resistance of cancer cells to radiotherapy treatment is usually common in patients with glioma, prostate cancer (PCa), and melanoma. Making it an ineffective treatment for this type of cancer (Kma, 2013; Chang et al., 2016).

The molecular mechanisms of intrinsic or acquired resistance of cancer cells are multifactorial; these mechanisms can range from altered expression of transport proteins, increased ability to repair DNA damage or the ability to copy your DNA even with mutagenesis-induced errors caused by the same targeted therapies, high tolerance to stress conditions, defects or evasion of apoptotic processes through senescence, alterations in oncogene/tumor suppressor expression and reprogramming of metabolic pathways (Rebucci and Michiels, 2013; Sarmento-Ribeiro et al., 2019; Cocetta et al., 2021).

Therefore, chemoresistance and radioresistance are challenges in the field of oncology that need to be further explored in order to be avoided. That is why researchers are currently studying new compounds that, combined with standard therapies, can improve their effectiveness, enhance their action, and, in turn, reduce the adverse effects of antineoplastic drugs to obtain better results in cancer treatment. In this sense, the natural compound resveratrol (RSV) is a good candidate for its anticancer properties, especially when combined with other chemotherapeutic drugs. For example, it has been reported that RSV can reduce the risk of multidrug resistance (MDR) through multiple cellular targets involved in carcinogenesis and chemo/radioresistance (Varoni et al., 2016; Ferraz da Costa et al., 2020). This review describes the molecular mechanisms by which RSV achieves its chemo- and radiosensitizing effects in cancer.

## 2 Chemistry and bioavailability of resveratrol

Many studies have demonstrated the chemopreventive effects of natural compounds such as curcumin, silymarin, allicin, lycopene, ellagic acid, and RSV. Furthermore, it has been reported that combining these natural compounds with anticancer drugs improves the anticancer activity of the drugs and reduces their side effects (Cragg and Pezzuto, 2016; Catanzaro et al., 2018; Berretta et al., 2020).

RSV (3,4′,5-trihydroxy-trans-stilbene) is found in many plants and foods such as grapes, blueberries, peanuts, berries, cocoa, etc. (Burns et al., 2002; Biesalski, 2007; Ko et al., 2017). It is a phytoalexin produced in plants as a defense mechanism in response to pathogen attacks (fungal or bacterial infections) or environmental stress (such as UV irradiation, metal salts, etc.) (Dercks and Creasy, 1989; Cocetta et al., 2021). RSV can be found in *cis* or *trans*-isomeric forms and their glycosides, trans-piceid and cis-piceid (Ali et al., 2010; Varoni et al., 2016; Ferraz da Costa et al., 2020). Since the publication by Jang *et al.* (Jang et al., 1997), the first article on the anticancer properties of RSV, the field of cancer research has given great interest to this molecule. In addition, a wide variety of beneficial biological effects of RSV have been discovered and explored, including its antioxidant, anti-inflammatory, anticancer, cardio- and neuroprotective activity (Park and Pezzuto, 2015; Kuršvietienė et al., 2016). However, *in vivo* experimental models have demonstrated that RSV is rapidly metabolized and eliminated, which leads to low bioavailability of the compound. Following oral administration, RSV is absorbed by passive diffusion or via membrane transporters at the intestinal level and is then released into the bloodstream, where it can be detected as an unchanged or metabolized molecule. (Ferraz da Costa et al., 2020). Even though 75% of RSV has been shown to be absorbed orally, only 1% is detected in the blood plasma after all metabolism (Varoni et al., 2016; Chimento et al., 2019; Ferraz da Costa et al., 2020).

To improve the bioavailability of RSV other means of RSV transport have begun to be used to enhance its bioavailability, such as delivering RSV through nanocarriers like nanoparticles or using different strategies, such as combining RSV with other compounds (bio-enhancers) (Kucinska et al., 2014; Santos et al., 2019; De Vries et al., 2021; Baek et al., 2023). For example, in the study of Zhang *et al.*, they developed a nanocarrier of RSV-loaded poly (ε-caprolactone)-poly (ethylene glycol) nanoparticles with an erythrocyte membrane. This system improved RSV’s poor water solubility and helped it escape the control of immune cells, improving its biocompatibility and tumor penetration *in vivo* models. Furthermore, they demonstrated for the first time that RSV could induce ferroptotic cell death in colorectal cancer by initiating lipid peroxidation and suppressing the expression of SLC7A11 and GPX4 (Zhang et al., 2022b).

Bioactive or bioenhancer compounds have also been used (piperine, quercetin, biflavone ginkgetin) that, in combination with RSV, improve bioavailability, solubility, absorption, and cellular permeability (De Vries et al., 2021; Jaisamut et al., 2021; Vesely et al., 2021).

Even in recent years, different synthetic derivatives of RSV (methoxylated, hydroxylated and halogenated), also known as prodrugs, have been developed to improve the bioavailability of RSV and its biological activities (Nawaz et al., 2017; Ferraz da Costa et al., 2020). Some examples of these are 3,5,4′-tri-O-acetyl-resveratrol (TARES) and resveratrol 3-O-β-D-glucopyranoside (De Vries et al., 2021).

On the other hand, some studies have reported that low daily doses of RSV have potent chemopreventive effects *in vivo* (Scott et al., 2012), which could be related to RSV conjugates or metabolites. Like many other polyphenols, RSV is metabolized by several enzymes, such as cytochrome P450 superfamily enzymes, sulfotransferases, and UDP-glucuronosyltransferases, to form conjugated (glucuronide and sulfated) metabolites. Unabsorbed polyphenols and their conjugates reach the lower gastrointestinal tract (cecum and colon) and interact with the intestinal microbiota. Dihydroresveratrol (DHR), lunularin (LUN), and 3,4′-dihydroxy-trans-stilbene are RSV metabolites derived from gut microbiota (Li et al., 2022). Interestingly, DHR and LUN have been shown to exert more potent antiproliferative and anti-inflammatory effects in renal and colonic cell lines, and it is suggested that DHR and LUN may contribute significantly to the chemopreventive properties elicited by RSV in the kidney and colon (Li et al., 2022).

## 3 Resveratrol as an anticancer compound

RSV has a wide variety of biological activities, such as antioxidant, anti-inflammatory, antiviral, neuroprotective, cardioprotective, immunomodulatory, and anticancer activity (Kotecha et al., 2016; Nawaz et al., 2017; Giordo et al., 2022). A large amount of literature reports the anticancer effects of RSV (Behroozaghdam et al., 2022; Wu et al., 2022; Zucchi et al., 2023).

**FIGURE 1:**
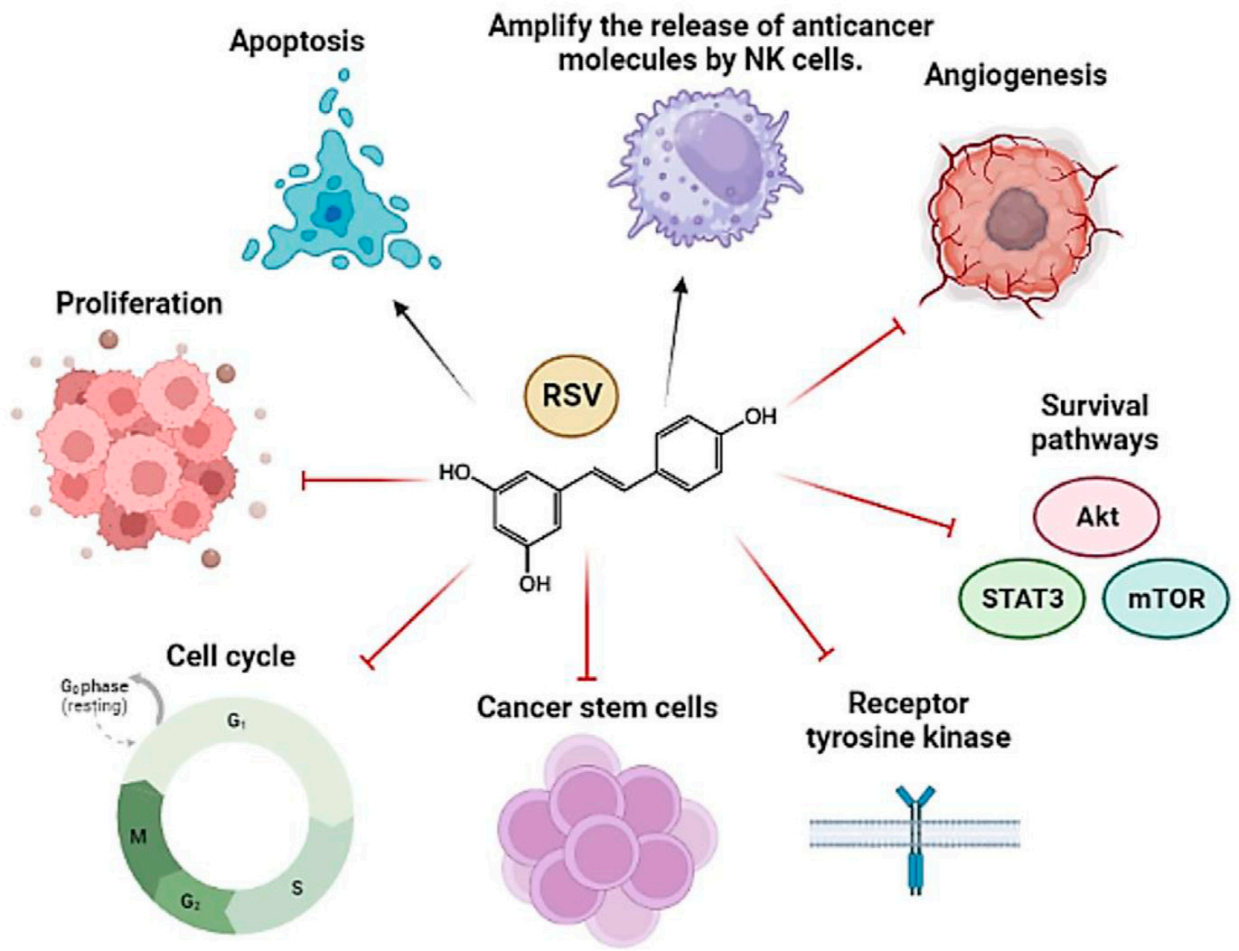
General representation of the main pathways and molecular targets affected by RSV in cancer. The arrows refer to promotion or increase, while the hammerhead lines refer to inhibition.

In fact, RSV exerts its antitumor effects through pleiotropic mechanisms of action (Repossi et al., 2020). Its ability to act on multiple targets has contributed to its usefulness as an anticancer agent (Varoni et al., 2016); in addition, in combination with other therapies (chemotherapeutics and radiotherapy, for example,), its ability to sensitize tumor cells resistant to such therapies has been demonstrated (El-Benhawy et al., 2021; Cheuk et al., 2022; Choi et al., 2022; Komorowska et al., 2022). Likewise, it has been seen that RSV protects healthy cells from the adverse effects of conventional agents (Ivanova et al., 2019). Therefore, its potential utility as an anticancer agent is quite attractive. Some RSV targets involved in the carcinogenesis process are exemplified in the following figure (Figure 1).

Due to its antioxidant and antimutagenic properties, RSV manages to prevent the onset of carcinogenesis. Likewise, it inhibits tumor growth and promotion by interfering with metabolic pathways such as glucose metabolism (Liu et al., 2018), inhibiting Cyclooxygenase-COX, and reducing the proliferative activity of cancer cells and their metastatic potential (Hu et al., 2016b). Furthermore, as shown by Schneider *et al.* (Schneider et al., 2001), RSV prevents the formation of colon tumors and reduces small intestine tumors in ApcMin/+ mice by decreasing the expression of genes directly involved in the progression of the cell cycle and cell proliferation (e.g., Cyclin D1 and D2). Moreover, RSV positively regulates genes activating immune cells such as HLA, FasL, and FOXP3, among others (Schneider et al., 2001; Fontenot et al., 2003; Ashrafizadeh et al., 2021).

On the other hand, several studies have shown the antitumor capacity of RSV by interfering with different signaling pathways such as STAT3, PI3K/Akt/mTOR, Wnt, insulin growth factor (IGF), SIRT1/AMPK, etc. (Habibie et al., 2014). In addition, RSV has also been reported to decrease NF-ĸB phosphorylation and acetylation, causing deficiencies in factors involved in tumor invasion and metastasis (I-CAM, AP-1, VEGF). The physical interaction between NF-ĸB and SIRT1 is involved in the anticancer activity of RSV (Buhrmann et al., 2016). SIRT1 inhibits NF-ĸB signaling by deacetylating the p65 subunit of the NF-ĸB complex. Furthermore, SIRT1 stimulates oxidative energy production by activating AMPK, PPARα, and PGC-1α, inhibiting NF-ĸB signaling and suppressing inflammation (Shao et al., 2015; He et al., 2017; Zhao et al., 2017). Also, RSV has been shown to suppress the growth of HCT116 colorectal cancer cells by inhibiting SIRT1-dependent NF-ĸB, in addition to inducing apoptosis in ls174t cells through the induction of the expression of the proapoptotic protein Bax inhibits the anti-apoptotic protein Bcl-2 (B-cell lymphoma 2) (Chen et al., 2009; Buhrmann et al., 2016).

In another critical study, Reagan-Shaw *et al.* (Reagan-Shaw et al., 2004) revealed that RSV significantly inhibits the induction of epidermal hyperplasia, mediated by exposure to UVB radiation through the decrease in proliferating cell nuclear antigen (PCNA), CDK-2, -4, and -6, as well as Cyclins-D1 and D2 in SKH-1 mouse cells. On the other hand, RSV in HaCaT cells inhibits cell proliferation by inhibiting the PI3K/AKT/mTOR pathway (Fabbrocini et al., 2010; Kisková and Kassayová, 2019). Another study reported that RSV suppresses cell growth and induces apoptosis in Colo16 squamous epidermal cancer cells (SCC) by inactivating Wnt and its target genes (survivin, c-Myc, cyclin D1, and VEGF). In addition, RSV increases the expression of the Wnt signaling inhibitor (Axin2) (Liu et al., 2017).

On the other hand, the role of resveratrol as an epigenetic regulator is very important in its anticancer activity. Let us remember that DNA hypermethylation (catalyzed by specific DNA methyltransferases (DMNT)) and histone deacetylation (mediated by histone deacetylases (HDACs) are key epigenetic mechanisms for the silencing and repression of many genes, including those involved in cell cycle regulation, DNA repair, inflammatory response, and apoptosis. Multiple studies have described the ability of RSV to increase or decrease the methylation of genes involved in tumorigenesis. For example, it has been shown that RSV can decrease the methylation of the promoters of the PTEN and BRCA-1 genes. Likewise, it has been seen that the methylation of the tumor suppressor gene RASSF1A and IL-10 decreases. And RSV can also increase the methylation of oncogenes such as AURKA, CCNB1, and HK2 (Lee et al., 2018). Additionally, RSV has also been involved in the deacetylation of genes such as p53 through SIRT1 (Lee et al., 2018; Rajendran et al., 2022).

Interestingly, it has also been reported that RSV modulates the expression of some miRNAs (short non-coding RNA) and lncRNAs (long non-coding RNA), which regulate the expression of genes involved in the malignant phenotype of cancer (Wang et al., 2019b; Asemi et al., 2023). For example, oncogenic miRNAs such as miR-19, miR-21, and miR-30a-5p have significantly decreased by RSV treatment in glioma (GBM) cells, modifying the expression of their target genes such as p53, PTEN, STAT3, NF-ĸB, COX-2 (Cocetta et al., 2021). On the other hand, lncRNAs have been identified as possible targets of RSV: MEG3 and ST7OT1 in GBM cell lines, U251 and U87, which increase with RSV treatment and induce apoptosis and necrosis of both cell lines. The MEG3 and ST7OT1 lncRNAs act as tumor suppressor genes. Ectopic expression of MEG3 and ST7OT1 inhibits cell proliferation and promotes apoptosis in human GBM cell lines (Wang et al., 2012; Liu et al., 2015). In contrast, RSV has been reported to decrease the expression of the lncRNA MALAT1 in colorectal and gastric cancer cells through the Wnt/β-catenin signaling pathway (Ji et al., 2014; Yang et al., 2018). The inhibition of MALAT1 expression by RSV is relevant as it is involved in the progression and metastasis of various types of cancer, including colorectal, gastric, lung, and hepatocarcinoma (Lai et al., 2012; Gutschner et al., 2013; Ji et al., 2014; Yang et al., 2018).

In a recent study, RSV in both its *cis* and *trans* forms was shown to inhibit the activity of the Anoctamin1 (ANO1) channel (a calcium-activated chloride channel, which is involved in the proliferation, migration, and invasion of various types of cancer, including head and neck squamous cell carcinoma, lung cancer, and prostate cancer) (Carter et al., 2014). In addition, they showed how RSV also decreased the expression of ANO1 protein and mRNA in PC-3 prostate cancer cells (Jeon et al., 2023).

## 4 Cancer and therapies

Cells constantly struggle with external stress and damage, which can result in mutations or severe cellular alterations if there is no successful repair. Usually, when there is very serious damage, the cell commits suicide to avoid further destruction and to eradicate genetically unstable and dangerous cells. However, if the cell death mechanism is not working properly, “malignant” cells can begin to proliferate, ultimately resulting in a tumor (cancer) (Zaitceva et al., 2021). Cancer remains one of the world’s leading causes of death, generating enormous costs and burdening humanity. The annual number of cancer cases worldwide is projected to increase from 19.3 million in 2020 to 30.2 million in 2040 (UICC Global Cancer Control, 2023).

The main goal of cancer treatment is the elimination of malignant cells through the induction of cell death. However, cancer cells constitute important barriers to clinical therapies due to their heterogeneity and plasticity. Resistance to cell death is one of cancer’s main characteristics, allowing the uncontrolled multiplication of cancer cells (Zaitceva et al., 2021). During the last years, several mechanisms have been described by which cancer cells can avoid cell death and acquire resistance to current treatments (surgery, radiotherapy, chemotherapy, targeted therapy, and immunotherapy). Among these are the overexpression of antiapoptotic proteins (Bcl-2) and the inactivation of p53 (Carneiro and El-Deiry, 2020). Oncologists point out that classical chemotherapy and radiotherapy are already reaching the limits of their effectiveness, so other methods or alternatives are needed to improve their effectiveness against cancer (Papież and Krzyściak, 2021).

In this review, we describe the signaling pathways and cellular mechanisms that lead to the development of chemoresistance and radioresistance in cancer cells.

Below is a table with information regarding the resistance that some types of cancer must radio and chemotherapy. It is worth mentioning that it is not yet known exactly which types of cancer will be resistant or sensitive to the therapies with which they will be treated since, as mentioned above, resistance can also be acquired during the treatment process, and multiple factors are involved in this. The development of resistance (tumor microenvironment, signaling pathways, cell-cell interactions, changes or mutations at the genetic and epigenetic level). However, this table was prepared based on articles and studies where emphasis is placed on the most studied specific types of cancer that tend to present or be more resistant to a specific therapy (Table 1).

**TABLE 1:** Mechanisms of resistance to chemo and radiotherapy in cancer.

Treatment	Resistant types of cancer	Resistance mechanisms	References
**Radiotherapy Chemotherapy whit temozolomide**	Glioblastoma cancer	Unmethylated MGMT (O6-methylguanine-DNA-methyltransferase) promoter	Mohammad et al. (2015), Gao et al. (2022)
		Overexpression of antiapoptotic proteins Bcl-2 and Bcl-xL	
		Inhibitors of apoptosis (IAP), such as XIAP, cIAP1, cIAP2, ILP2, ML-IAP, and surviving	
		Overexpression of lncRNA (PDIA3P1)	
**Chemotherapy whit temozolomide**	Melanoma	Activating mutations of BRAF serine/threonine kinase	Mohammad et al. (2015), Zhai et al. (2020)
		Overactivation of the MAPK and PI3K/AKT pathways	
		Overexpression of the positive regulator of Bcl-2, NF-κB	
		Activation of NLRP1 inflammasomes	
**Chemotherapy whit 5-fluorouracil**	Colorectal cancer	Deregulation of Wnt, Notch, Hedgehog and/or TGF-β signaling pathways involved in the proliferation and maintenance of CSCs	Blondy et al. (2020)
		Overexpression of FasL that triggers Fas-mediated apoptosis of T cells	
		High expression and greater activity of some membrane drug transporters (MDR) such as MRP8/ABCC11, ABCC5, MRP7/ABCC10, and ABCB1	
**Chemotherapy whit carboplatin, paclitaxel**	Ovarian cancer	Overexpression of the alpha 1 chain of collagen type I (COL1A1)	Zhang et al. (2021b), Yang et al. (2021)
		Upregulation of drug resistance protein CSAG2 by cytoplasmic polyadenylation element binding protein 4 (CPEB4)	
**Chemotherapy whit paclitaxel, doxorubicin Radiotherapy**	Breast cancer	Hypermethylation of the Krüppel-like factor 4 (KLF4) promoter by DNA-methyltransferase 1 (DNMT1)	Gilreath et al. (2021), Xiang et al. (2021), Lin et al. (2022)
		P-glycoprotein protein can pump doxorubicin out of MCF-7 cells	
		Overexpression of breast cancer resistance protein (BCRP)	
		A hypoxic microenvironment that promotes resistance to radiotherapy	
**Radiotherapy**	Prostate cancer	Alteration of the DNA damage repair system, cell cycle disorders, imbalance of redox homeostasis, EMT, CSC and hypoxia in the tumor nucleus	Lyu et al. (2023)

## 5 Signaling pathways and mechanisms leading to chemoresistance and radioresistance

The characteristics of cancer cells that promote resistance to therapies, currently more described, are the following. Some cancer cells are said to resemble stem cells, defined as cancer stem cells (CSCs); These cells frequently change during tumor progression and after therapeutic exposures, favoring their resistance and progression (Basu et al., 2022). Tumors have also been shown to harbor a population of slow-cycling cells (SCCs) that are not in the proliferative cell cycle and are inherently refractory to antimitotic drugs. However, they can stochastically re-enter the proliferative cell cycle or respond to mitogenic stimuli. Like CSCs, SCCs can evade the immune system and survive cancer treatments, thereby influencing treatment failure, tumor recurrence, and metastasis. Unlike CSCs, SCCs represent a population of transient cells that haphazard go in and out of the G0/G1 phase very quickly (Basu et al., 2022).

On the other hand, a group of cancer cells acquires the ability to resist therapies through anastasis. Anastasis is, in a few words, the arrest of apoptosis and the ability that cells acquire to maintain themselves in a state of senescence after treatment. After this, they preserve and increase their proliferative capacity, making anastasis undesirable during cancer therapy (Zaitceva et al., 2021). Cancer cells enter the anastasis process because some of the mitochondria of these cells remain intact during the apoptosis process; this is by increasing the levels of Bcl-2/Bcl-xL, which inhibit the process of permeabilization of the outer membrane of mitochondria (MOMP). Allowing these mitochondria to repopulate in the cell (Peña-Blanco and García-Sáez, 2018). A limited number of mitochondria undergo MOMP; the amount of cytochrome c released is insufficient to trigger apoptosis but sufficient for sublethal activation of caspase and consequent activation of endonuclease, leading to genome instability (Zaitceva et al., 2021).

On the other hand, polyploid giant cancer cells (PGCC) are found in several types of cancers and have been seen to play an important role in resistance to treatments such as radiotherapy and chemotherapy. These cells are characterized by having multiple nuclei or a single giant nucleus with multiple complete sets of chromosomes. In addition, they contribute to the immortality, invasion, and metastasis of tumors. For example, Was *et al.* showed how polyploidy develops in response to various genotoxic stresses, such as chemotherapy, radiation, hypoxia, oxidative stress, or environmental factors such as air pollution, ultraviolet light, or hyperthermia (Liu et al., 2022; Was et al., 2022). The general mechanism leading to PGCC formation could be a consequence of endoreplication, which is related to genetic or physical disorders of mitosis, cell fusion, or cell cannibalism. An important evolutionary feature of polyploid cancer cells is the generation of aneuploid clones during depolyploidization, which expands cancer cells’ genetic repertoire, allowing them further development and expansion (Mittal et al., 2017; Liu et al., 2022; Pienta et al., 2022; Was et al., 2022).

**FIGURE 2:**
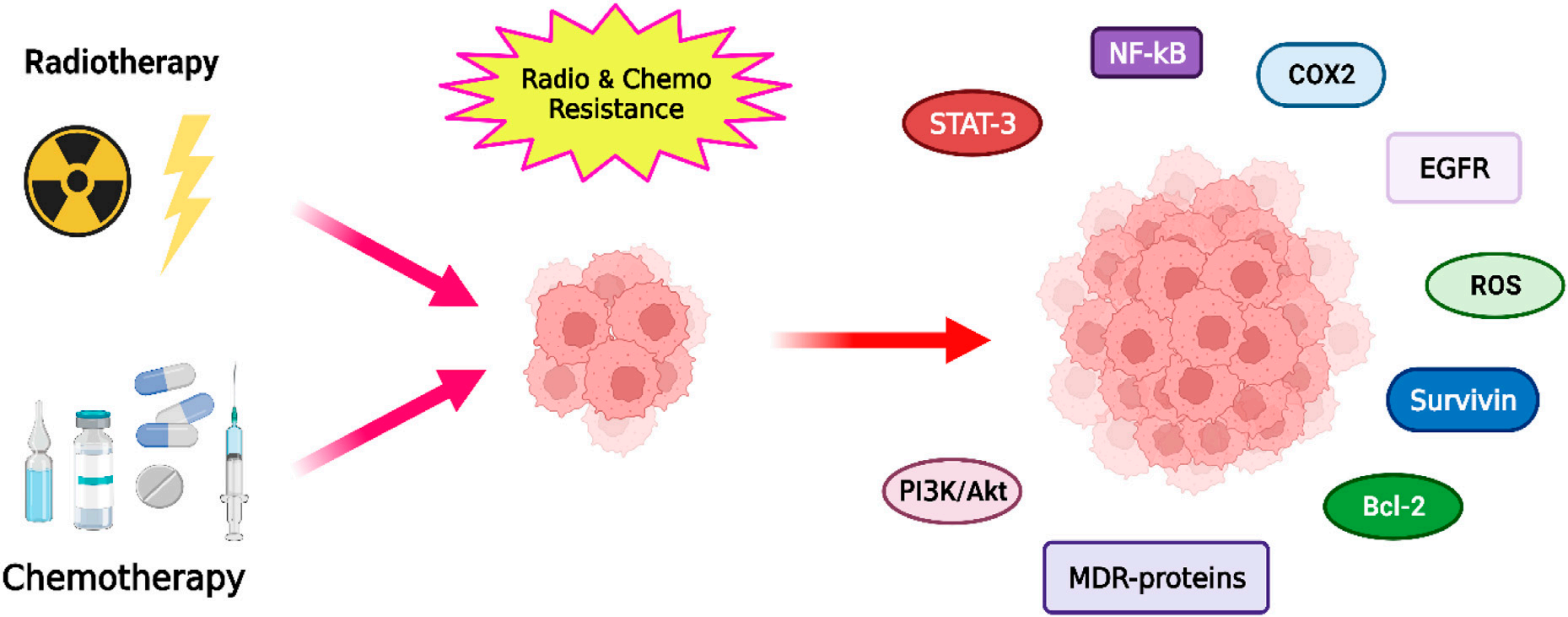
Representation of the main pathways, factors and proteins involved in the development of radioresistance and chemoresistance in cancer cells.

Chemoresistance and radioresistance mechanisms in tumor cells can be intrinsic (cells are resistant before treatment) or acquired (resistance develops during treatment). Below, we describe some of the most important mechanisms and signaling pathways (Figure 2), but for a more detailed review, you can consult the works of Fodale et al., 2011; Chang et al., 2016; Mashouri et al., 2019; Ali et al., 2020; Zaitceva et al., 2021; Fodale et al., 2011; Chang et al., 2016; Mashouri et al., 2019; Ali et al., 2020; Zaitceva et al., 2021).

### 5.1 Reactive oxygen species (ROS) and TNF

Members of the tumor necrosis factor (TNF) superfamily are important inducers of apoptosis, contributing to the death response in tumor cells. However, they are also involved in developing adverse reactions such as resistance to anticancer drugs; these effects are mediated by the production of ROS (Garg and Aggarwal, 2002; Blaser et al., 2016; Cruceriu et al., 2020).

The release of TNF stimulates the activation of the vascular endothelium, release of nitric oxide, recruitment of inflammatory cells, immunoglobulins, complement and alters the permeability of the mitochondrial membrane, favoring the release of cytochrome C and the subsequent activation of caspases, which, in turn, leads to apoptosis. Furthermore, TNF signaling is directly related to the mitochondrial electron transport mechanism and ROS production (Chandel et al., 2001). ROS are an upstream component that activate the process of TNF-induced apoptosis, followed by caspases, mitogen-activated protein kinases (MAPKs), NF-κB, and activation of the transcription factor AP-1 (Garg and Aggarwal, 2002). Oxidative stress is essential in tumor development and cancer therapy (Gorrini et al., 2013). It has been reported that ROS can promote oncogenic mutations and epigenetic changes in cancer cells. These alterations lead to the loss of tumor suppressor genes, accelerated cell metabolism, and altered cell sensitivity to anticancer drugs (Pelicano et al., 2004; Panieri and Santoro, 2016; Priya et al., 2017).

ROS are generated by redox-sensitive pro-survival signaling pathways (oxidation-reduction reactions), which function as intermediates in the transduction of various extracellular signals (Vinod et al., 2013). However, there is a complex intracellular redox network to protect cells against oxidative stress, where several signaling pathways can be activated in the adaptive response of ROS (glucose metabolism, PI3K/Akt pathway, MAPK pathway). These signaling pathways play a critical role in protecting cancer cells against the cytotoxic effects of antineoplastic agents, leading to chemoresistance (Marengo et al., 2019). For example, in chemoresistant epithelial ovarian cancer (EOC) cells, an increase in ROS and apoptosis was observed with the combination of Dactolisib (BEZ235) and cisplatin treatment, which also inhibited the PI3K/Akt/mTOR signaling pathway, reversing epithelial-mesenchymal transition (EMT) and decreasing CSC marker expression compared to cisplatin monotherapy (Deng et al., 2019).

On the other hand, ROS-mediated genotoxic stress has been shown to be involved in NaAsO_2_ -induced cell cycle arrest, decreased stemness, and chemoresistance of prostate cancer cells (PC-3 and DU145) (Zhang et al., 2021c).

### 5.2 NF-κB signaling pathway

NF-κB is activated by different molecules and signaling pathways, and in turn, this transcription factor modulates the expression of several genes (more than 500 genes) that participate in inflammatory responses, cell differentiation, adaptation to stress, apoptosis, immunity, and one of the main effector pathways that regulate the amount of ROS that leads to cell survival and at the same time to chemoresistance in cancer (Morgan and Liu, 2011; Vinod et al., 2013).

Among the many diseases related to aberrant NF-κB activation, cancer has been the main focus due to the role of NF-κB as a central regulator of the regulation of genes involved in cell survival (Bcl-2, Bcl-xL, inhibitors of apoptosis proteins [IAP] and superoxide dismutase) (Garg et al., 2005) and tumor progression (intercellular adhesion molecule 1 [ICAM1], vascular cell adhesion molecule 1 [VCAM1], leukocyte-endothelial adhesion molecule 1 [PECAM- 1], vascular endothelial growth factor [VEGF], hypoxia-inducible factor [HIF-1], Cyclooxygenase-2 [COX-2], inducible nitric oxide synthase [iNOS], and matrix metalloproteinase [MMP-9]). Furthermore, NF-κB activity is frequently elevated in many tumor types, including leukemia, lymphoma, prostate cancer, breast cancer, colon cancer, melanoma, and head and neck cancer (Garg et al., 2003; Dolcet et al., 2005; Fan et al., 2013; Wu et al., 2017).

NF-κB can be aberrantly activated in cancer by chemotherapeutic agents and ionizing radiation, response to stress, and induced cell death, leading to treatment-induced resistance of tumor cells (Jung and Dritschilo, 2001; Garg et al., 2003; Sampepajung et al., 2021). *In vitro* and *in vivo* studies have shown that NF-κB inhibits chemotherapy-induced apoptosis in various tumor types (Bharti and Aggarwal, 2002; Sampepajung et al., 2021). For example, the upregulation of NF-κB-inducible genes has been shown to protect MDA MB-231 breast cancer cells from apoptosis induced by paclitaxel and ionizing radiation (Newton et al., 1999). Similarly, in another study, they treated cell lines of different types of cancer [Hep-3B (liver), AGS (gastric), SiHa (cervical), MCF7 (breast), NTUB1 (bladder), and H460 (lung-non-small cells)] with different chemotherapy regimens (doxorubicin, 5-fluorouracil [5-FU], cisplatin, and paclitaxel), and found a correlation between cell survival with the level of drug-induced NF-κB activity (Chuang et al., 2002). On the other hand, in a study where the inhibitory subunit of NF-κB was transfected, an increase in chemotherapeutic efficacy was observed *in vitro* and *in vivo* models of gastrointestinal neoplasms (Cusack et al., 2000).

Another interesting study is that of Park M. *et al.* (Park et al., 2016); they evaluated the role of protein tyrosine kinase 7 (PTK7) in ESCC resistance to radiotherapy. They observed that PTK7 plays an important role in ESCC radioresistance through activation of the NF-ĸB pathway. In addition, these authors reported an increase in the IAPs, XIAP, and survivin, encoded by NF-ĸB regulated genes, which was associated with radioresistant cells but not in radiosensitive cells; nevertheless, PTK7 knockdown downregulated IAP expression (Park et al., 2016).

### 5.3 COX-2

COX-2 is an enzyme expressed primarily in response to inflammatory disorders and cancer. It is responsible for mediating the production of prostaglandins and is under clinical investigation as a target for cancer therapy (Milas, 2001; Nakata et al., 2004; Gowda et al., 2017). Over-expression and activity of COX-2 have been associated with more aggressive tumor phenotypes and worse prognosis in patients with breast, colon, head, neck, lung, and pancreatic cancer (Wang et al., 2014; Li et al., 2020). Furthermore, evidence suggests that COX-2 is involved in multiple aspects of carcinogenesis, including tumor growth, metastatic spread, and resistance to various therapies (Harris, 2009; Tong et al., 2018). As a result, some scientists have investigated the usefulness of selective COX-2 inhibitors, such as SC-236 and Celecoxib, *in vitro* and *in vivo* to test whether their inhibition can sensitize tumor cells to make treatment more efficient against cancer using chemotherapy and radiotherapy. For example, SC-236 increased the response to radiotherapy in various murine tumor models (you can put here which tumor types) and in a human GBM xenograft in nude mice (Kishi et al., 2000; Petersen et al., 2000). On the other hand, the combination treatment with celecoxib and imatinib resulted in a significant decrease in cell viability and an increase in caspase-3 enzyme activity in HT-29 colon cancer cells (Atari-Hajipirloo et al., 2016).

In another study, celecoxib and afatinib co-treatment inhibited the expression of COX-2 and EGFR, which led to increased sensitization of A549 lung cancer cells to radiotherapy and apoptosis (Zhang et al., 2021a).

### 5.4 Bcl-2 and p53 mutant

The development of resistance to cell death mechanisms, specifical death by apoptosis, is one characteristic that distinguishes tumor cells and plays an important role in developing resistance against anticancer agents.

Bcl-2 is an anti-apoptotic protein that is overexpressed in several solid and hematopoietic tumors, and that also exerts its influence by improving cell survival (Kaufmann and Vaux, 2003), which contributes to resistance to conventional treatments, including chemotherapy and radiotherapy (Deng et al., 2000; Maji et al., 2018). In addition, several studies have shown that Bcl-2 inhibition sensitizes tumor cells to chemotherapy and radiotherapy. For example, transfection with the PTEN gene, which negatively regulates Bcl-2, potentiated the effects of radiation therapy on several prostate cancer cell lines (PC-3-Neo, PC-3-Bcl-2, and LNCaP) (Rosser et al., 2004). Another study that used an antisense oligonucleotide against Bcl-2 observed an increase in apoptosis and greater chemotherapeutic efficacy in a thyroid carcinoma cell line (Kim et al., 2003). These results and others have highlighted Bcl-2 as a potential target for chemosensitization and radiosensitization (Belka and Budach, 2002; Gutiérrez-Puente et al., 2002). For example, in the study carried out by Lu L *et al.* (Lu et al., 2018), in the MB-468 radioresistant breast cancer cell line, they observed low levels of ROS and higher levels of STAT3 and Bcl-2 proteins; on the other hand, when they added Niclosamide, a potent STAT3 inhibitor, radioresistance was overcome by inhibiting STAT3 and Bcl-2 and inducing ROS (Lu et al., 2018).

On the other hand, the tumor suppressor protein p53 is a key factor in inducing cell cycle arrest, DNA repair, and apoptosis in response to cellular stress. Unfortunately, it is known that in approximately more than 50% of cancerous tumors, p53 is mutated (Levine, 1997). Consequently, there is increased survival and proliferation of cancer cells. In addition, there is evidence suggesting that inactivation of the p53 wild-type protein results in increased chemo-resistance to several chemotherapeutic drugs, including doxorubicin, cisplatin, 5-fluorouracil (5-FU), and etoposide (Ferraz da Costa et al., 2012).

Moreover, the mutant p53 gain of function can induce chemoresistance, increasing drug efflux and metabolism, survival promotion, apoptosis inhibition, upregulation of DNA repair, autophagy suppression, microenvironmental resistance elevation, and the induction of cancer stem cells (He et al., 2017). However, recently, a subset of cancer cells, regardless of their p53 status, exhibited resistance to chemotherapy through the p21 protein. p21 is a transcriptional target of p53 that is induced upon DNA damage and acts to arrest the cell cycle by inhibiting cyclin-dependent kinases (CDKs) (Ashraf et al., 2019).

Hsu *et al.* Investigate the proliferation-senescence decision in response to chemotherapy and elucidate how early p21 dynamics predict and shape cell fate. They concluded that cells with high or low levels of p21 during doxorubicin treatment are destined to become senescent, while those with an intermediate amount of p21 proliferate after drug washout (Ashraf et al., 2019; Hsu et al., 2019). This data is undoubtedly important for stimulating senescence in the context of cancer therapies.

### 5.5 Survivin

Survivin is part of the mammalian IAP family, and its main function is to inhibit the apoptosis pathway by blocking the activation of caspases 3, 8, and 9 (Salvesen and Duckett, 2002). Both *in vitro* and *in vivo* experiments have shown cancer-inducing properties of surviving (Bao et al., 2002), as well as overexpression in various types of cancer and absence in most normal tissues (Altieri, 2003). Survivin expression has been shown to increase in VEGF-stimulated vascular endothelial cells (O’Connor et al., 2000; Mesri et al., 2001; Tran et al., 2002). In addition, high levels of surviving expression have been associated with a high rate of tumor recurrence, poor overall patient survival, and high tumor resistance to chemotherapy and radiotherapy in several cancers, including lung, breast, colon, stomach, esophagus, pancreas, liver, uterus, and ovary cancer among others (Altieri, 2003). Nestal de Moraes *et al.* (Nestal de Moraes et al., 2015) demonstrated that the transcription factor FOXM1 upregulates the expression of the anti-apoptotic genes XIAP and Survivin, which contributes to the development of drug resistance and is associated with poor clinical outcomes in breast cancer patients (Nestal de Moraes et al., 2015).

In contrast, survivin inhibition has been reported to sensitize breast cancer cells to paclitaxel, etoposide, doxorubicin, and cisplatin (O’Connor et al., 2000; Wall et al., 2003; Mita et al., 2008; Lyu et al., 2018; Minaiyan et al., 2021); furthermore, survivin inhibition in combination with radiotherapy resulted in a significant decrease in lung cancer cells survival (Lu et al., 2004).

### 5.6 Multidrug resistance (MDR) proteins or carrier proteins

Since the antitumor agent must reach the cancer cell in an adequate concentration to exert its effect, drug uptake or release alterations could also be responsible for the acquisition of chemoresistance (Huang and Sadée, 2006). Transport proteins, also called ATP-dependent multidrug transporters (ABCs), associated with chemoresistance are multidrug resistance proteins (MDR1; P-glycoprotein [P-gp]; MRP1; ABCB1; ABCC1) (Leonard et al., 2003; Crouthamel et al., 2006; Pérez-Gutiérrez et al., 2007), the multidrug resistance-associated protein (MRP1) (Hong et al., 2019), the protein related to lung resistance (LRP) (Schneider et al., 2001; Wang, 2011) and breast cancer resistance protein (BRCP) (Fu et al., 2020). MDR proteins are ATP-binding proteins that regulate P-gp, which are responsible for removing drugs from cells using ATP hydrolysis (Yang et al., 2014).

Many drugs, including daunorubicin (DRN), imatinib, nilotinib, taxol, and doxorubicin, among others, can be expelled from cancer cells that overexpress P-gp and multidrug resistance-associated protein 1 (MRP1) transporter (Gottesman et al., 2002; Kosztyu et al., 2014). For example, in one study, high expression and activity of MRP1 were observed in primary cultures of glioblastoma multiforme biopsies (Quezada et al., 2011). Also, in PC3 and DU145 human prostate cancer cell lines, increased expression of MRP1 in prostate cancer cells is related to resistance to chemotherapy. Similarly, the blockade of MRP1 function by leukotriene receptor antagonists (MK-571 and zafirlukast) led to an intracellular accumulation of the MRP1 substrate and increased sensitivity to cytotoxic drugs (van Brussel and Mickisch, 2003). This result was consistent with the study where they used an NF-ĸB inhibitor to inhibit MDR protein expression, leading to increased apoptosis in prostate cancer cells (Flynn et al., 2003).

On the other hand, elevated levels of ABCB1 have also been shown to be associated with paclitaxel resistance in human osteosarcoma (OS) cell lines, which developed cross-resistance with other ABCB1 substrates, such as doxorubicin, docetaxel, and vincristine (Yang et al., 2014). In addition, the involvement of ABCB1 overexpression in doxorubicin resistance in human OS cells was demonstrated by downregulation or abrogation of ABCB1 expression, which resulted in the restoration of doxorubicin sensitivity (Fanelli et al., 2016; Liu et al., 2016; Serra et al., 2021).

Moreover, the study by Ranibar S *et al.* (Ranjbar et al., 2019) used compounds derived from 5-oxo-hexahydroquinoline that they named D6, D5, and D3 (which have 3-chlorophenyl, 2,3-dichlorophenyl and 4-chlorophenyl substituents in the C4 position of the 5-oxo-hexahydroquinoline core) these compounds inhibit P-gp, MRP1, and BCRP, respectively; causing a reversal of drug resistance (Doxorubicin, mitoxantrone) at concentrations of 1–10 μM, in human uterine sarcoma cells (MES-SA) sensitive and resistant to drugs with P-gp overexpression.

### 5.7 AKT signaling pathway

Protein kinase B (PKB or Akt) is a downstream effector of PI3K and has been described as a mediator of anti-apoptotic signaling in cancer cells. In addition, Akt overexpression has been shown to promote cell cycle progression and tumorigenesis (Zhan and Han, 2004). Akt may contribute to chemoresistance and radioresistance: for example, over-expression of Akt1 has been reported to result in increased resistance of lung cancer cells (describe which cell lines) against a panel of various chemotherapeutic agents (doxorubicin, cisplatin, and mitoxantrone) (Hövelmann et al., 2004). Accumulating evidence to date has suggested that the PI3K/Akt pathway may also be an essential contributor to radioresistance (Zhan and Han, 2004). In this regard, Akt activation in bile duct cancer cells has been shown to be associated with radioresistance, which was demonstrated through indirect inhibition of Akt activation with a PI3K inhibitor (LY294002) (Tanno et al., 2004).

On the other hand, in a recent study, the PI3k/Akt/mTOR pathway was associated with the increase and activation of PDK1, which is associated with radioresistance, motility, and invasiveness of hepatocellular carcinoma. Furthermore, it was observed that pharmacological inhibition of PDK1 in Huh7 cells mediated by BX795 synergistically enhances the radiosensitivity of these cells, increases the apoptotic Bax/Bcl-2 ratio, and abolishes oncogenicity and clonogenicity (Bamodu et al., 2020).

Another study showed that the inactivation of AKT signaling inhibited tumorigenesis and radioresistance mediated by CPNE1 in triple-negative breast cancer cells. Knockdown of CPNE1 also inhibited tumor growth and promoted cell apoptosis *in vivo* in mouse xenografts (Shao et al., 2020).

In addition, the participation of the PI3K/AKT/mTOR pathway in chemoresistance has also been reported. For example, in the study by Qiu C *et al.* (Qiu et al., 2020), they show that MNAT1, a cyclin-dependent kinase-activating kinase (CAK) complex, contributes to OS cell resistance to cisplatin via the PI3K/AKT/mTOR. MNAT1 is highly expressed in various types of cancer and is involved in the molecular pathogenesis of cancer and drug resistance (Qiu et al., 2020).

### 5.8 STAT3

STAT3 is a member of the STAT family of transcription factors that are activated by tyrosine phosphorylation through signaling mediated by receptors such as epidermal growth factor receptor (EGFR), platelet-derived growth factor (PDGF), and cytokines such as interleukin-6 (IL-6) (Akira, 2000; Levy and Darnell, 2002). It is established that IL-6 is produced in an autocrine or paracrine manner and plays an essential role in the malignant progression of various types of cancer, including multiple myeloma (MM), by regulating the growth and survival of tumor cells. The presence of IL-6 leads to constitutive activation of STAT3, resulting in the expression of high levels of the anti-apoptotic protein Bcl-xL (Catlett-Falcone et al., 1999; Bharti et al., 2004). Therefore, STAT3 has various biological functions, including regulation of cell growth, apoptosis, and cell differentiation. STAT3 has also been shown to be permanently active in various human cancers and is required for tumor cell proliferation (Bromberg, 2001). Furthermore, it has been reported that STAT3 can mediate chemoresistance, and its inhibition can sensitize cells to apoptosis. For example, in the study by Bharti *et al.* (Bharti et al., 2004), inhibition of STAT3 contributed to decreased survival of multiple melanoma cells and sensitized pancreatic cancer cells to apoptosis (Greten et al., 2002). Blockade of STAT3 using various techniques sensitized breast cancer cells (MDA-MB435) to apoptosis induced by taxol and adriamycin (doxorubicin) chemotherapy (Real et al., 2002). Other studies also show that STAT3 inhibition increases radiosensitivity in different tumors such as hepatocellular carcinoma, squamous cell carcinoma of the head and neck, gastric cancer, pancreatic cancer, *etc.*, (Adachi et al., 2012; Bu et al., 2013; Huang et al., 2014; 2016; Lee et al., 2019; You et al., 2019; Wang et al., 2020b).

### 5.9 EGFR signaling pathway

EGFR is a transmembrane glycoprotein with intrinsic tyrosine kinase activity. By binding with EGF, it regulates a signaling cascade that, in turn, regulates cell growth and proliferation. Similarly, it activates molecular pathways involved in various cellular processes, such as cell differentiation, survival, and transformation (Yarden and Sliwkowski, 2001). EGFR overexpression has been related to more aggressive tumor phenotypes, poor patient prognosis, and lack of response to antitumor therapies (Wang, 2017). For example, increased EGFR expression is associated with increased tumor chemoresistance and radioresistance in tumors, including squamous cell carcinoma, ovarian adenocarcinoma, hepatocarcinoma, glioblastoma, and adenosquamous carcinoma of the cervix (Akimoto et al., 1999; Milas et al., 2000; Nasu et al., 2001; Wang et al., 2020a). On the other hand, various investigators have also reported increased sensitization of tumor cells to radiotherapy through EGFR inhibition in head and neck squamous cell carcinoma (SCC), human colon cancer (GEO), colon cancer, ovarian (OVCAR-3), glioblastoma multiple (Huang et al., 1999; Bianco et al., 2000; Huang and Harari, 2000; Wang et al., 2020a).

Further studies have confirmed that the EGFR/PI3K signaling pathway plays an important role in tumor chemoresistance (Zhang et al., 2019b). showed that p53 sensitized cisplatin-chemoresistant NSCLC (Non-small cell lung cancer) by suppressing the EGFR/PI3K signaling pathway. Similarly, when miR-7 inhibited the EGFR/PI3K signaling pathway, adriamycin sensitivity in breast cancer (MCF-10 and MCF-7/ADR) increased (Huang et al., 2019).

### 5.10 Glutathione/glutathione S transferase system

It has also been reported that chemoresistance may be mediated by the glutathione/glutathione S transferase (GSH/GST) system (Zhu et al., 2006). An essential function of GSH is the detoxification of xenobiotics and some endogenous compounds, maintaining intracellular redox balance. These substances are electrophilic and form conjugates with GSH, either spontaneously or enzymatically, in reactions catalyzed by GSH-GSTs (Traverso et al., 2013). Several studies have shown a relationship between the resistance of tumor cells to chemotherapy drugs and an increase in the expression of GSH, GST, and GPx (Chao et al., 1992; Buser et al., 1997).

In contrast, low levels of GSH, GST, and GPx have been found to be associated with favorable clinical features and a good prognosis. In contrast, high GSH and GST activity levels were associated with more aggressive or more advanced disease in tissue samples from women with breast cancer (Buser et al., 1997). In fact, cancer cell lines containing low levels of GSH are much more sensitive to ionizing radiation than cells that overexpress GSH (Meister, 1991).

Increased GSH is an important factor contributing to drug resistance by binding or reacting with drugs, interacting with ROS, preventing protein or DNA damage, or participating in DNA repair processes. For example, GSH depletion and GGT inhibition in melanoma cells significantly increased cytotoxicity through oxidative stress (Benlloch et al., 2005). In addition, cells that overexpress GGT have been shown to be more resistant to hydrogen peroxide and to drugs such as doxorubicin (Hochwald et al., 1997), cisplatin (Godwin et al., 1992), and 5-fluorouracil (McLellan and Wolf, 1999).

### 5.11 DNA repair

Direct or indirect alterations in DNA are the basis of the mechanism of action of many drugs used in cancer therapy. Therefore, increased DNA repair activity compromises the damage induced by chemotherapeutic agents, resulting in chemoresistance (Sakthivel and Hariharan, 2017). Tumor cells have obtained a great capacity to repair damaged DNA through multiple pathways, such as mismatch repair (MMR), base excision repair (BER), nucleotide excision repair (NER), repair by non-homologous end joining (NHEJ), homologous recombination (HR) repair among others. For example, the BER repair pathway is involved in colon cancer resistance to temozolomide chemotherapy (Liu et al., 1999). The “excision repair cross-complement protein 1” (ERCC1), which belongs to the NER repair pathway, has also been reported to be associated with chemoresistance to platinum-based anticancer agents of various tumors, including cancer lung, colon, and breast cancer (Youn et al., 2004).

On the other hand, the Werner syndrome protein (WRN), a DNA helicase vital for the regulation/activation of NHEJ and HR repair, as well as the maintenance of DNA telomere stability, is found to be overexpressed in glioblastoma multiforme cancer cells and is also associated with increased resistance to chemotherapeutic agents, especially cisplatin (Lee et al., 2016). It has also been reported that the ectopic expression of the HOTAIR protein (antisense RNA of the HOX transcript), an important regulator of the transcription factor NF-ĸB, is associated with a more significant DNA damage response to cisplatin treatment and also a greater chemoresistance in ovarian cancer cells (IGROV, OVSAHO, OVMUNA, SKOV3, A2780, HEYC2, A2780-CR5, and OV90) (Özeş et al., 2016). Otherwise, it has been reported that BRCA1, another important protein in homologous recombination repair, activates NF-ĸB in response to topoisomerase inhibitor drugs such as etoposide or camptothecin; NF-ĸB, for its part, transcriptionally activates anti-apoptotic proteins such as Bcl-2 and XIAP (X-linked inhibitor of apoptosis), thus causing chemoresistance (Harte et al., 2014).

### 5.12 Proteasomal pathway

The proteasome participates in the degradation of marked proteins that are no longer necessary for the cell or proteins that have suffered some damage or modification by ubiquitination. It also modulates the levels of proapoptotic, antiapoptotic, growth regulatory and stress response factors. Alterations in this proteolytic system are associated with various pathologies, including cancer. Inhibition of proteasome activity results in the upregulation of proapoptotic factors, such as p53, Bax, and Noxa, while reducing the levels of antiapoptotic proteins, such as Bcl-2 and IAP family proteins (McConkey and Zhu, 2008; Vinod et al., 2013).

Proteasome inhibitors have been shown to promote apoptosis in various types of cancer and induce sensitivity to combination chemotherapeutic agents. For example, bortezomib, an inhibitor of the proteasomal pathway, plays an important role in combination chemotherapy with lenalidomide and thalidomide in MM by stimulating the immune system, inhibiting angiogenesis, and sensitizing cancer cells, thereby overcoming chemoresistance (Orlowski, 2004; Vinod et al., 2013; Piechotta et al., 2019).

### 5.13 Hypoxia

Hypoxia is a common feature of all solid neoplasms (Rankin and Giaccia, 2016; Chouaib et al., 2017; Najafi et al., 2020); cellular responses to hypoxia are usually regulated by the family of factors hypoxia-inducible factor (HIF) transcriptions (Harris, 2002; Keith and Simon, 2007). HIF is a protein complex formed by a heterodimer consisting of an HIFα subunit and a HIFβ subunit (Li et al., 2009). Under normoxic conditions, the Von Hippel-Lindau tumor suppressor gene product ubiquitinates HIFα and degrades it at the proteasomal pathway, but in hypoxia, the interaction between HIFα and VHL is abolished. As a result, HIFα is stabilized, dimerizes with HIFβ, and then binds to hypoxia-responsive elements in the promoters of hypoxia-regulated genes (Li et al., 2009). The HIF dimer activates gene transcription that modulates cell survival, proliferation, metabolism, and angiogenesis (Harris, 2002; Li et al., 2009; Schwab et al., 2012). Hypoxia is related to a poor prognosis (van den Beucken et al., 2014; Carnero and Lleonart, 2016; Qin et al., 2017); Hypoxia during tumorigenesis can develop by two mechanisms: chronic or acute hypoxia. Chronic hypoxia occurs due to the high proliferation of cancer cells; therefore, they are constantly expelled from the blood vessels. In contrast, acute hypoxia is caused by a temporary cessation of blood flow due to poor tumor vasculature (Brown and Wilson, 2004). Regardless of the mechanism, tumor hypoxia has been widely documented as contributing to resistance to all anticancer therapies, including chemotherapy and radiotherapy (Brown and Wilson, 2004; Mitani et al., 2014; Jeong et al., 2019; Najafi et al., 2020).

Indeed, silencing or inhibition of HIF-1 increases radiation sensitivity in various tumor models. For example, HIF-1 knockdown in human hepatoma cells inhibits proliferation, induces apoptosis, and promotes radiosensitivity in chemically induced hypoxia (Yang et al., 2011; Wang et al., 2019a). In prostate cancer cell lines, the knockdown of HIF-1 by siRNAs induces apoptosis and cell cycle arrest at the G2/M transition, resulting in radiosensitization (Huang et al., 2012, 201). In xenograft tumors with FaDu (Hypopharyngeal carcinoma) and ME180 (Squamous cell carcinoma) cell lines, blockade of the HIF1 response during transient hypoxic stress increases hypoxia, reduces lactate levels, and improves response to high doses of single fraction radiation (Leung et al., 2017; Wang et al., 2019a). In laryngeal carcinoma, the inhibition of HIF-1α and glucose transporter-1 (GLUT1) expression increases radiosensitivity and promotes apoptosis and necrosis (Shen et al., 2017; Wang et al., 2019a).

Many HIF-1-inducible genes, such as VEGF, Glut-1, MDR, IAP3, and Bcl-2, directly or indirectly mediate chemoresistance (Liu et al., 2008; Doktorova et al., 2015). In various types of tumors, such as hepatocellular carcinoma, neuroblastoma, and lung cancer, HIF-1α inhibition re-sensitizes cells to drug treatment; therefore, it is considered a valid target to reduce drug resistance induced by reverse hypoxia (Liu et al., 2008; Sullivan et al., 2008; Huang et al., 2010; Hartwich et al., 2013; Doktorova et al., 2015).

## 6 Resveratrol as a radiosensitizing agent

Radiation therapy (XRT) is a cancer treatment that uses high doses of radiation to kill cancer cells. It has been widely used in breast cancer (Moon et al., 2009), prostate cancer (Zietman et al., 2010), carcinoma lung (Machida et al., 2003), medulloblastoma or glioblastoma (Habrand and De Crevoisier, 2001), melanoma (Ivanov et al., 2007), etc.

Ionizing radiation (IR) promotes its effects by inducing DNA damage and activating DNA damage-induced signaling pathways (Kma, 2013). These pathways result in cell cycle arrest or induction of cell death by apoptosis, necrosis, autophagy, or mitotic catastrophe, depending on the total dose (Wang et al., 2006; Suit et al., 2007; Kma, 2013). However, the efficacy of XRT is limited by the radioresistance exhibited by cancer cells (Leone et al., 2008; Ruan et al., 2009; Yang et al., 2012). For instance, prostate cancer is highly resistant to IR (Crook et al., 1995; Hagan et al., 2000). The doses of XRT that are usually used in the treatment of prostate adenocarcinoma are up to 70 Gy and have shown biochemical failure rates of 30% or more, which leads to the need to increase the dose of XRT, which, in turn, results in impotence, rectal and bladder toxicity (Zietman et al., 2010). Likewise, it has been reported that the increase in radiation dose leads to the incidence of skin toxicity in patients undergoing XRT (Weiss and Landauer, 2003). These effects add to some of the side effects of XRT, such as pituitary hormone dysfunction, behavioral problems, and reduced neurogenesis (Baskar et al., 2012; Michaelidesová et al., 2019), which consequently shows diminished therapeutic outcome and poor quality of life for survivors.

Therefore, researchers have focused on finding drugs or compounds that function as effective radiosensitizers, reducing the radiation dose-response threshold for cancer cells with minimal side effects in normal cells (Kma, 2013).

In this context, it has been reported that RSV has radioprotective effects; thanks to its antioxidant properties, this compound acts as a scavenger of free radicals or ROS. In addition, it has been reported that it reduces inflammation by inhibiting IL-8 expression and blocking NF-κB activation (Benitez et al., 2009; Oh et al., 2009). The radioprotective effects of RSV *in vivo* were possibly first demonstrated in the study by Carsten *et al.* (Carsten et al., 2008), where it was shown that RSV in combination with IR resulted in a reduction in the frequency of total chromosomal aberrations in mouse bone marrow cells, compared to untreated, RSV-only treated groups hear. In this case, mice were administered RSV at a dose of 100 mg/kg body weight per day, started 2 days before whole body irradiation with 3 Gy (at a dose rate of 1.18 Gy)/min., and analyzed 1 and 30 days after irradiation. It was evident from this observation that RSV possesses a potential radioprotective property (Carsten et al., 2008; Kma, 2013).

On the other hand, several studies have shown that using RSV in combination with radiotherapy increases therapeutic efficacy against cancer (Fang et al., 2012; 2013; Komorowska et al., 2022). The following table exemplifies some studies that have seen the radiosensitizing effect of RSV and the mechanisms involved in it (Table 2).

**TABLE 2:** Radiosensitizing effect of RSV in cancer cells.

Type of cancer	Doses	Results	References
**Glioblastoma (GBM) - derived radioresistant tumor- GBM-CD133 cells**	Radiation doses of 2, 4, 6, 8, and 10 Gy	Radiosensitization was induced through STAT3 inhibition and increased apoptosis	Yang et al. (2012)
	With RSV (100 µM)		
**U87 GBM cells**	Radiation dose of 5 Gy	The combined treatment demonstrated a significant increase in the arrest of cancer cells in the S phase after irradiation, compared to RSV or radiation alone	Leone et al. (2008)
	With RSV (20 µM)		
**U87 GBM cells**	Radiation dose of 2 Gy	RSV inhibited HIF-1α, in addition to decreased colony formation and increase DNA damage in GBM cells. And in combination with IUdR there is increased radiosensitization	Khoei et al. (2016)
	With RSV (20 µM)		
	And Iododeoxyuridine (IUdR) (1 µM)		
**Breast cancer cells (MCF-7)**	Radiation dose of 4 Gy	RSV + IR combination treatment has been shown to trigger a cascade of events leading to suppressing p53 and p53 signal transduction genes by NF-κB inhibition, ultimately leading to cell death	Aravindan et al. (2013)
	With RSV (100 µM)		
**Prostate cancer cells (PCa, PC3, and 22RV1)**	Radiation dose of 2 Gy	RSV can reverse radioresistance by inhibiting Akt phosphorylation, inducing the expression of antiproliferative molecules (p53, p21cip1, and p27kip1), increasing IR-induced expression of markers of DNA damage (γH2Ax) and apoptosis (caspase 3)	Rashid et al. (2011)
	With RSV (5 µM)		
**C6 (C3031)- GBM** **Rats inoculated with C6 cells for 2 weeks**	Radiation dose of 5 Gy	RSV enhanced radiation modulation of inflammation, cell cycle, and apoptosis. In addition, DNA damage was attenuated, and cell arrest was induced in the G0/G1 phase of GBM rats, accompanied by changes in the expression of proteins related to the ATM-AKT-STAT3 pathway	Qian et al. (2022)
	With RSV (40 mg/kg)		
**Radioresistant prostate cancer cells (PC-3)**	Radiation dose of 2, 4, 6, 8 Gy	RSV has a radiosensitizing effect by reducing the expression of cancer stem cell markers and affecting EMT markers	El-Benhawy et al. (2021)
	With RSV (35, 70, 140 µM)		
**Lung adenocarcinoma cells (A549)**	Radiation dose of 6 Gy	RSV combined with irradiation treatment decreased the expression of STIM1 and Orai1. RSV sensitizes A549 cells and significantly enhances the effect of irradiation damage	Lele et al. (2021)
	With RSV 10–200 µM)		

**FIGURE 3:**
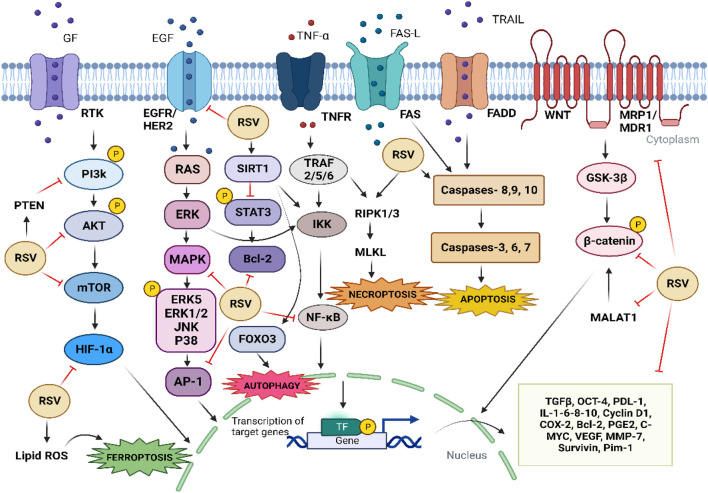
Mechanisms by which RSV contributes to chemosensitization and radiosensitization of cancer cells. The arrows refer to activation, while the hammerhead lines refer to inhibition.

In addition to the mechanisms mentioned above, it has been reported that RSV can induce senescence, apoptosis, autophagy, and inhibition of DNA repair, as well as the ability to kill cancer stem cells more efficiently, leading to radiosensitization of cancer cells (Figure 3) (Luo et al., 2013; Wang et al., 2015).

## 7 Chemosensitization of tumor cells by resveratrol

Chemosensitization is based on using a drug or compound that enhances the activity of another by influencing one or more resistance mechanisms, making it a valuable strategy to overcome the chemoresistance developed by cancer cells. In addition, it dramatically reduces the adverse effects that occur due to the toxicity of high doses of drugs used in cancer treatment.

*In vitro* and *in vivo* studies show that RSV can reverse chemoresistance in tumor cells by modulating apoptosis and downregulation of drug transporters and proteins involved in cancer progression (Figure 3) (Lee et al., 2016).

### 7.1 Paclitaxel and resveratrol

Paclitaxel is one of the drugs used in chemotherapy to treat different types of cancer, including ovarian, breast, and lung lymphoma, among others. Some side effects after treatment with paclitaxel are anemia, bruising, bleeding, nausea, diarrhea, tingling in the hands and feet, tiredness, hair loss, muscle pain, etc. (Weaver, 2014).

The primary mechanism behind RSV chemosensitization to paclitaxel chemotherapy is the downregulation of Bcl-2 and MDR1/P-gp family members. It has been suggested that RSV-mediated inhibition of the ERK1/2 and AP-1 pathways leads to decreased Bcl-xL in non-Hodgkin’s lymphoma and MM cell lines (Cusack et al., 2000; Jazirehi and Bonavida, 2004).

In another study, when RSV was administered prior to paclitaxel treatment in lung cancer cell lines (A549, EBC-1, Lu65), a significant improvement in the antiproliferative potential of paclitaxel was observed. Furthermore, RSV also caused cell cycle arrest in the G1 and G1/S phases of the cell cycle by inducing the expression of the CDK inhibitors p21/WAF1/CIP1 and p27/KIP1, allowing the dose of paclitaxel required to kill tumor cells to be reduced (Shankar et al., 2007).

Downregulation of survivin is another mechanism by which RSV enhances proliferation-inhibitory effects through S-phase cell cycle arrest and increased apoptosis in neuroblastoma cells treated with paclitaxel, in addition to other drugs such as doxorubicin, cytarabine, taxol, actinomycin, and methotrexate (Fulda and Debatin, 2004). RSV has also been reported to decrease survivin expression in a dose-dependent manner in a multidrug-resistant human non-small cell lung cancer cell line (SPC-A-1/CDDP). Similarly, in a more recent study, RSV was found to increase the sensitivity of renal cells (Caki-1) resistant to paclitaxel by suppressing survivin expression (Min et al., 2019).

Another study demonstrated the synergistic interaction of RSV and paclitaxel in inducing apoptosis in the DBTRG glioblastoma cell line. In this work, they observed that the combination with RSV increases apoptosis markers such as mitochondrial membrane depolarization, ROS levels, and caspase 3 activity in DBTRG cells, compared to treatment with paclitaxel alone. The synergistic effect seems to be mediated by the stimulation and activation of the TRPM2 channel sensitive to mitochondrial oxidative stress, which allows Ca^2+^ to enter cancer cells, contributing to their death (Öztürk et al., 2019; Cocetta et al., 2021).

### 7.2 Doxorubicin/adriamycin/hydroxydaunorubicin and resveratrol

Doxorubicin (adriamycin or hydroxydaunorubicin) is a drug (anthracycline-type antibiotic) with antitumor activity produced by *Streptococcus peucetius* var. *caesius*; used to treat different types of cancer, such as leukemia, lymphoma, neuroblastoma, sarcoma, Wilms tumor, lung, breast, stomach, ovary, thyroid, and bladder cancer. It can intercalate with DNA, inhibiting DNA and RNA synthesis. It induces a cell cycle arrest during the S phase (Rivankar, 2014). In several studies it has been seen that RSV can sensitize DOX-resistant cancer cells, such as those shown below.

Fenig *et al.*; found that RSV treatment decreases MRP1 expression in AML cells resistant to doxorubicin. Furthermore, when RSV was administered, the expression of MRP1 decreased, while the cellular uptake of DOX in resistant cells increased. Based on these observations, the authors concluded that RSV might facilitate cellular DOX uptake through the downregulation of MRP1 and that RSV may help to overcome DOX resistance or sensitize AML cells resistant to doxorubicin (Fenig et al., 2004; Gupta et al., 2011). Likewise, RSV chemosensitization to DOX is mediated by inhibition of MDR1/P-gp and Bcl-2 in ovarian cancer cells (OVCAR-3) (Rezk et al., 2006), acute myeloid leukemia (AML-2) (Kweon et al., 2010) and oral squamous cell carcinoma (KBv200) (Quan et al., 2008).

Also, RSV has been shown to increase the chemosensitivity of tumor cells by arresting the cells at different stages of the cell cycle. For example, in DOX-chemoresistant B16 melanoma cells, RSV increased DOX-induced cytotoxicity and decreased cyclin D1 expression. In addition, DOX treatment combined with RSV was associated with an increased cell cycle arrest in the G1/S phase (Gatouillat et al., 2010).

On the other hand, in breast cancer cells (MCF-7/adr) and MDA-MB-231 resistant to DOX, it has been reported that the combination of DOX with RSV inhibits cell growth, promotes apoptosis, and suppresses cell migration (Cocetta et al., 2021). The effect of RSV is linked to the modulation between SIRT1 and β-catenin; RSV was shown to be able to increase SIRT1 (deacetylase) levels and decrease β-catenin expression by ubiquitination, which reversed chemoresistance (Jin et al., 2019). Further experiments showed that RSV treatment significantly increased cellular accumulation of DOX by decreasing the expression levels of the ATP-binding cassette (ABC) transporter genes, MDR1 and MRP1 in MCF-7/adr and MDA-MB-231 cells., as well as in a xenograft model (*in vivo*) revealing that RSV and DOX treatment in combination significantly inhibits tumor volume (Breast cancer) by 60%, compared to the control group (Kim et al., 2014).

Furthermore, in the study by (Mitani et al., 2014), they investigated the effect of RSV on hypoxia-induced doxorubicin resistance in MCF-7 cells. They were observing how RSV and its derivative 3,5-dihydroxy-4′-methoxy-trans-stilbene, reverse hypoxia-induced doxorubicin resistance at a concentration of 10 μM through the decrease in HIF protein expression -1α and HIF-1 activity activated by hypoxia. Similarly, RSV inhibited the expression of CBR1 induced by hypoxia at the mRNA and protein levels (Mitani et al., 2014).

The combination of DOX and RSV also increases Bax gene expression in HCT116 colon cancer cell lines; furthermore, RSV enhances intracellular DOX uptake by blocking P-gp activity, thereby sensitizing colorectal cancer cells to DOX (Khaleel et al., 2016).

Also, it has been reported that the acquisition of DOX resistance in SGC7901 gastric cancer cells may be due to EMT induced by aberrant activation of Akt, giving cancer cells the ability to overexpress genes related to DOX chemoresistance. This cellular model showed that RSV reverses DOX resistance by suppressing EMT by inhibiting the PI3K/Akt signaling pathway, activating caspase-3-dependent apoptosis. In addition, RSV induced cell cycle arrest by increasing PTEN expression in addition to suppressing cell invasion and N-cadherin expression (Xu et al., 2017).

On the other hand, RSV-inducing chemosensitivity in breast cancer cells (MCF-7) resistant to adriamycin or DOX has also been shown to be dependent on miR-122-5p inhibition. Moreover, inhibition of miR-122-5p showed a significant effect on the regulation of critical anti-apoptotic proteins such as Bcl-2 and cyclin-dependent kinases (CDK2, CDK4, and CDK6) in breast cancer cells (MCF-7) in response to RSV (Zhang et al., 2019a).

In a recent study, Moreira *et al.* demonstrated that RSV increases the expression of the SIRT1 gene in LoVo cells (derived from metastatic colon adenocarcinoma nodules). SIRT1 negatively regulates the expression of survivin, a major inhibitor of apoptosis and which, as seen earlier in this review, is involved in the resistance of cancer cells to chemotherapeutic therapies. They demonstrated that increased expression of the SIRT1 gene contributed to overcoming resistance to apoptosis in DOX-resistant LoVo colon cancer cells (Moreira et al., 2022). In the study by Xiong Le *et al.*, they prepared nanoparticles with sustained release capacity and targeted IL-13Rα2 to improve its bioavailability from the RSV. These nanoparticles were inserted into an ATC/anaplastic thyroid cancer mouse model, demonstrating that RSV effectively inhibits ATC growth *in vivo*. And that it can overcome the resistance to DOX and Docetaxel in this model (Xiong et al., 2021).

### 7.3 Temozolomide (TMZ) and resveratrol

Temozolomide is a chemotherapy drug that mostly treats brain tumors (GBM, medulloblastomas, neuroblastomas, and sarcomas). Temozolomide comes in capsules and can be used alone or in combination with XRT. It is an alkylating cytostatic agent that, when is activated, forms free radicals capable of causing DNA degradation and even single and double-strand DNA breaks that induce cell cycle arrest in G2/M, which eventually leads to cell apoptosis (Yan et al., 2016). The increase in resistance to TMZ is one of the main reasons for the failure of glioblastoma treatment (Perazzoli et al., 2015; Lee, 2016).

RSV has been shown to enhance the therapeutic efficacy of TMZ in several ways. One proposed mechanism is the reduction of autophagy mediated by an increase in ROS, favoring apoptosis. In the glioblastoma multiforme (GBM) cell line SHG44, TMZ, in combination with RSV, markedly increased the production of ROS, which served as a signal for the activation of AMP-activated protein kinase (AMPK). Subsequently, activated AMPK inhibited mTOR signaling and decreased levels of the anti-apoptotic protein Bcl-2, contributing to the additive antiproliferative effects of combined TMZ and RSV treatment. These results were also confirmed *in vivo* mouse models (GBM orthotopic xenograft), where the combination of TMZ and RSV treatment induced a reduction in tumor volume and tumor proliferation, which was associated with decreased expression of Ki −67, a proliferation index marker (Yuan et al., 2012). Other studies indicate that GBM-initiating cells (GIC), which display stem cell properties, are involved in tumor resistance to TMZ, and RSV has been shown to enhance GIC sensitivity to TMZ by activating the pATM/pATR/p53 pathway and promoting cancer cell apoptosis. In addition, this work demonstrated that RSV inactivated p-STAT3, promoting the differentiation of glioblastoma-initiating cells (Li et al., 2016). RSV has recently been seen to sensitize glioma cell lines with strong resistance to TMZ through the inhibition of Wnt2 and β-catenin and increased expression of GSK-3β (Yang et al., 2019).

### 7.4 Cisplatin and resveratrol

Cisplatin or CDDP (alkylating agent) is a drug widely used in cancer that inhibits DNA synthesis by producing crosslinks within DNA chains known as adducts; its cytotoxic activity is produced by binding to all DNA bases, with a preference for guanine and adenosine bases. It is widely used to treat testicular, ovarian, bladder, head and neck, esophageal, small cell and non-small cell lung, breast, cervical, stomach, prostate, Hodgkin and non-Hodgkin lymphoma, neuroblastomas, sarcomas, MM, melanoma and mesothelioma (Galanski, 2006).

In addition to the high cytotoxicity of cisplatin, the main limitation of the clinical utility of this drug against cancer is the high incidence of chemoresistance (Galluzzi et al., 2012). Below are some studies where RSV sensitizes and improves the effectiveness of treatment with cisplatin.

RSV and cisplatin together show more effective inhibition of non-small lung cancer cell (NSCLC) proliferation and induction of apoptosis than cisplatin treatment alone (Cocetta et al., 2021). In fact, in the study by Ma L *et al.* (Ma et al., 2015), it was shown that the combination of cisplatin and RSV dramatically improved the efficacy of cisplatin in depolarizing the mitochondrial membrane potential, increasing the release of cytochrome C, as well as decreasing Bcl-2 expression and increased Bax protein in cisplatin-resistant H838 and H520 non-small lung cancer cells, resulting in increased inhibition of proliferation and induction of apoptosis (Ma et al., 2015). Synergistic effects of RSV in combination with cisplatin have also been shown in A549 lung carcinoma cells. The results show that the combination favors autophagy by lessening autophagosome accumulation, AKT phosphorylation, and LC3-II protein levels (Hu et al., 2016a).

The impact of combined RSV and cisplatin treatment has also been tested in hepatocarcinoma cells; Liu and others 2018 showed that RSV-induced chemosensitivity to cisplatin is associated with an imbalance in redox homeostasis that favors DNA damage and apoptosis. The data indicate that RSV can inhibit glutamine metabolism of resistant human hepatocarcinoma cell lines (C3A and SMCC7721), increasing the toxic effect of chemotherapy but not on normal liver cells (Liu et al., 2018; Cocetta et al., 2021).

Another interesting study is that of Weiguo (Zhao et al., 2010), who analyzed the efficacy of RSV with non-small cell lung cancer cells or NSCLC (SPC-A-1/CDDP) resistant to multiple drugs (Paclitaxel, taxol, gefitinib, cisplatin, etc.). Cells were treated with RSV at a concentration of 25, 50, or 100 μM *in vitro* studies, and tumors were induced in nude mice implanted with SPC-A-1/CDDP cells and fed a special diet containing included RSV at a dose of 1 g/kg/day or 3 g/kg/day. In this work, they evaluated cell proliferation, apoptosis, the distribution of cell cycle phases, the IC50 values of cisplatin, gefitinib, and paclitaxel, the volume of the implanted tumor, and the expression of survivin in mice treated with RSV compared to the control. RSV significantly inhibited the proliferation of NSCLC cells, induced apoptosis, arrested the cell cycle between G0-G1 and S phases or in the G2/M phase, and decreased IC50 values of chemotherapeutic drugs (cisplatin, gefitinib, and paclitaxel) (Gupta et al., 2011). Furthermore, RSV showed antitumor effects in mice, affecting tumor proliferation in a dose- and time-dependent manner; similarly, survivin expression in SPC-A-1/CDDP cells decreased after RSV treatment (Zhao et al., 2010).

### 7.5 Oxaliplatin and resveratrol

Oxaliplatin or OXA (bifunctional alkylating agent) is a type of platinum chemotherapy that binds covalently to DNA and forms platinum-DNA adducts to inhibit DNA replication and transcription (Kelland, 2007). The intrastrand crosslinks formed by OXA can block DNA replication and transcription (Woynarowski et al., 2000). OXA is used to treat colorectal cancer and, in some cases, throat cancer (esophagus) (Kelland, 2007). However, the development of resistance to OXA *in vitro* and *in vivo* in colon cancer has been reported (Hsu et al., 2018).

Kaminski *et al.* (Kaminski et al., 2014) investigated the effect of RSV on the antitumor activity of oxaliplatin in the resistant colon cancer cell line Caco-2 and its possible involvement in the inflammatory response. The results showed that the combined treatment synergistically inhibits cell growth and induces apoptosis with caspase-3 activation, PARP cleavage, and mitochondrial membrane depolarization (Cocetta et al., 2021). In addition, primary macrophages derived from human monocytes were seeded and differentiated to add later supernatants of tumor cells (Caco-2) treated with RSV/OXA and the co-cultures were maintained for 24 h, noting how the co-treatment prevents the immunosuppression of the co-cultured macrophages, making them potentially tumoricidal (Kaminski et al., 2014).

In addition, RSV prevents OXA-induced neuronal damage and peripheral neuropathic pain (CIPNP), which is a common and devastating side effect of cancer therapy. Avoiding the upregulation of NFκB, TNFα, ATF3, and c-fos, increasing the expression of Nrf2, NQO-1, HO-1, and the redox-sensitive deacetylase SIRT1 (Recalde et al., 2020).

### 7.6 5-Fluorouracil (5-FU) and resveratrol

5-FU is a drug that inhibits the activity of thymidylate synthetase, the enzyme responsible for producing Thymidine, acting as an antimetabolite, inducing cell cycle arrest, and promoting apoptosis (Longley et al., 2003). 5-FU has shown the most significant impact in treating colorectal cancer (CRC), although it has also been used in treating breast, stomach, pancreas, and certain types of skin cancer (Gu et al., 2019). Despite its great advantages, the clinical application of 5-FU is limited due to the development of resistance of cancer cells. In fact, there is increasing evidence showing that cancer stem cells (CSC) present in the tumor microenvironment (TME) are the main ones responsible for resistance to 5-FU (Yang et al., 2015; Das et al., 2020).

Several works have shown the efficacy of RSV in potentiating the cytotoxic effect of 5-FU (Moutabian et al., 2022). A study conducted in colorectal cancer cells (HCT116 and DLD1) showed that the combined treatment increases cell cycle arrest and decreases the proliferation and migration of colorectal cancer cells by inhibiting the PI3K/Akt signaling pathway. Likewise, RSV showed anti-inflammatory effects by inhibiting pSTAT3 and NFκB proteins (Chung et al., 2018). The combination of RSV and 5-FU also inhibits the proliferation and migration of B16 murine melanoma cells by decreasing the levels of AMPK, COX-2, VASP, and VEGF, compared to the compounds alone (RSV and 5-FU) (Lee et al., 2015). In addition, Buhrmann and others 2018 demonstrated that RSV could reduce TNF-β-induced survival and migration of resistant HCT116 colorectal cancer cells by promoting 5-FU sensitization (Buhrmann et al., 2018).

Recently, Brockmueller *et al.* demonstrated in tumor microenvironments of 5-FU-resistant HCT-116 and HCT-116R colorectal cancer (CRC) cells with 3D alginate and monolayer cultures how RSV increased the sensitivity of CRC cells to 5-FU by reducing vitality, migration, proliferation, angiogenesis, invasion, and epithelial-mesenchymal transition (Brockmueller et al., 2023).

### 7.7 Gemcitabine and resveratrol

Gemcitabine (2′, 2′-difluoro 2′-deoxycytidine) is a drug that induces cancer cell death by inhibiting ribonucleotide reductase, an enzyme necessary for the synthesis of deoxyribonucleotides, in addition to inhibiting DNA polymerase. Gemcitabine inhibits DNA synthesis; the cells cannot divide properly and die. Gemcitabine is used to treat different types of cancer, including carcinoma of the bladder, pancreas, oral squamous cell carcinoma, non-small cell lung, ovary, and breast (Mini et al., 2006).

Similarly, it has been shown that cytochrome p450 1b1 (Cyp1b1) is overexpressed in many neoplasms and plays an important role in developing resistance to chemotherapy. Interestingly, RSV has been reported to downregulate Cyp1b1, thereby increasing apoptosis induced by antimetabolites such as 5-FU and gemcitabine in gemcitabine-chemoresistant cholangiocarcinoma (Mz-ChA-1, HuCC-T1, CCLP1, and SG231) tumor models. Mitomycin C and 5-FU, although the precise mechanism of RSV-mediated chemosensitization associated with Cyp1b1 inhibition is unknown (Frampton et al., 2010). However, it has been shown in an orthotopic mouse model of human pancreatic cancer that RSV enhanced the antitumor activity of gemcitabine, and this was associated with decreased expression of Bcl-2, Bcl-xL, COX-2, cyclin D1, MMP-9 and VEGF (Harikumar et al., 2010).

### 7.8 Docetaxel and resveratrol

Docetaxel is an antineoplastic drug that stabilizes microtubules, inhibiting their polymerization, which causes cell death by interruption of mitosis. Docetaxel is used to treat different types of cancer, including breast cancer, prostate, non-small cell lung cancer (NSCLC), stomach, ovary, bladder, soft tissue sarcoma, melanoma, and head and neck cancer (Rodríguez Carranza, 2015). Docetaxel resistance in breast cancer cells (SK-BR-3, MCF7, MDA-MB-231, and T47D) is associated with HER2 expression.

HER-2 is a receptor of the epidermal growth factor family involved in cell growth and development. In addition, it promotes the recruitment of several proteins, which lead to the activation of signal transduction cascades such as: the PI3K/AKT/mTOR and RAF/MEK/ERK pathways. Overexpression of this receptor contributes to the progression and survival of breast cancer (Fink and Chipuk, 2013; Li and Li, 2013; Cocetta et al., 2021). Interestingly, RSV treatment inhibits HER-2 activation through docetaxel-induced blockade of MAPK and Akt signaling, as well as survival signaling pathways activated by HER-2, enhancing the sensitization of breast cancer cells (SK-BR-3 and MDA-MB-231) to docetaxel (Vinod et al., 2015).

In a recent study, EGF-conjugated hybrid lipid polymer nanoparticles (LPN) were fabricated to co-deliver docetaxel (DTX) and RSV in non-small cell lung cancer (NSCLC). *In vitro* and *in vivo* studies demonstrated that EGF DTX/RSV LPNs have significant synergistic effects, the best tumor inhibition capacity, and the lowest systemic toxicity. These results suggest that EGF DTX/RSV LPNs may be a promising strategy for treating and chemosensitization NSCLC (Song et al., 2018). Similarly, Zhang et al. designed PEGylated nanoliposomes to co-deliver Docetaxel and RSV in Balb/c nude mice bearing prostate cancer (PC3), demonstrating the efficiency of the treatment as a whole (Zhang et al., 2022a). Other recent studies of RSV as a chemo-sensitizer are summarized in Table 3.

**TABLE 3:** Chemo-sensitizing effects of resveratrol.

Treatment	Type of cancer	Effects	References
**Adriamycin + Resveratrol and quercetin Polymeric micelles (nanostructures) Pluronics®**	Resistant ovarian cancer xenograft models	They reduce cardiotoxicity induced by adriamycin and, at the same time, act as chemosensitizers	Fatease et al. (2019)
**Cisplatin + Resveratrol**	Breast cancer Cisplatin-resistant MDA-MB-231 (cisR)	It improved chemosensitivity by inhibiting IL-6 production and STAT3 activation and reversing macrophage polarization	Cheuk et al. (2022)
**Gemcitabine + Resveratrol**	Pancreatic cancer: MiaPaCa-2 and Panc-1 And KPC mouse model	RSV inhibited lipid synthesis through SREBP1. This decreased the sphere-forming ability and suppressed the expression of CSC markers	Zhou et al. (2019)
**Temozolomide + Resveratrol**	Glioblastoma: A172 and LN428 cells	RSV negatively regulated STAT3, inhibited cell proliferation and migration, and induced apoptosis, accompanied by elevated levels of its negative regulators: PIAS3, SHP1, SHP2, and SOCS3. Combined therapy reversed the TMZ resistance of LN428 cells, which could be related to the decreased levels of O6-methylguanine-DNA methyltransferase (MGMT) and STAT3	Wu et al. (2023)
**Cisplatin + Resveratrol**	Breast cancer Cisplatin-resistant MCF-7	RSV decreases cisplatin resistance and induces serine 20 (S20) phosphorylation on p53. Activate p53 target genes such as PUMA and Bax, restoring apoptosis. Bcl-2 decreased, and Bax protein increased	Hernandez-Valencia et al. (2018)

**FIGURE 4:**
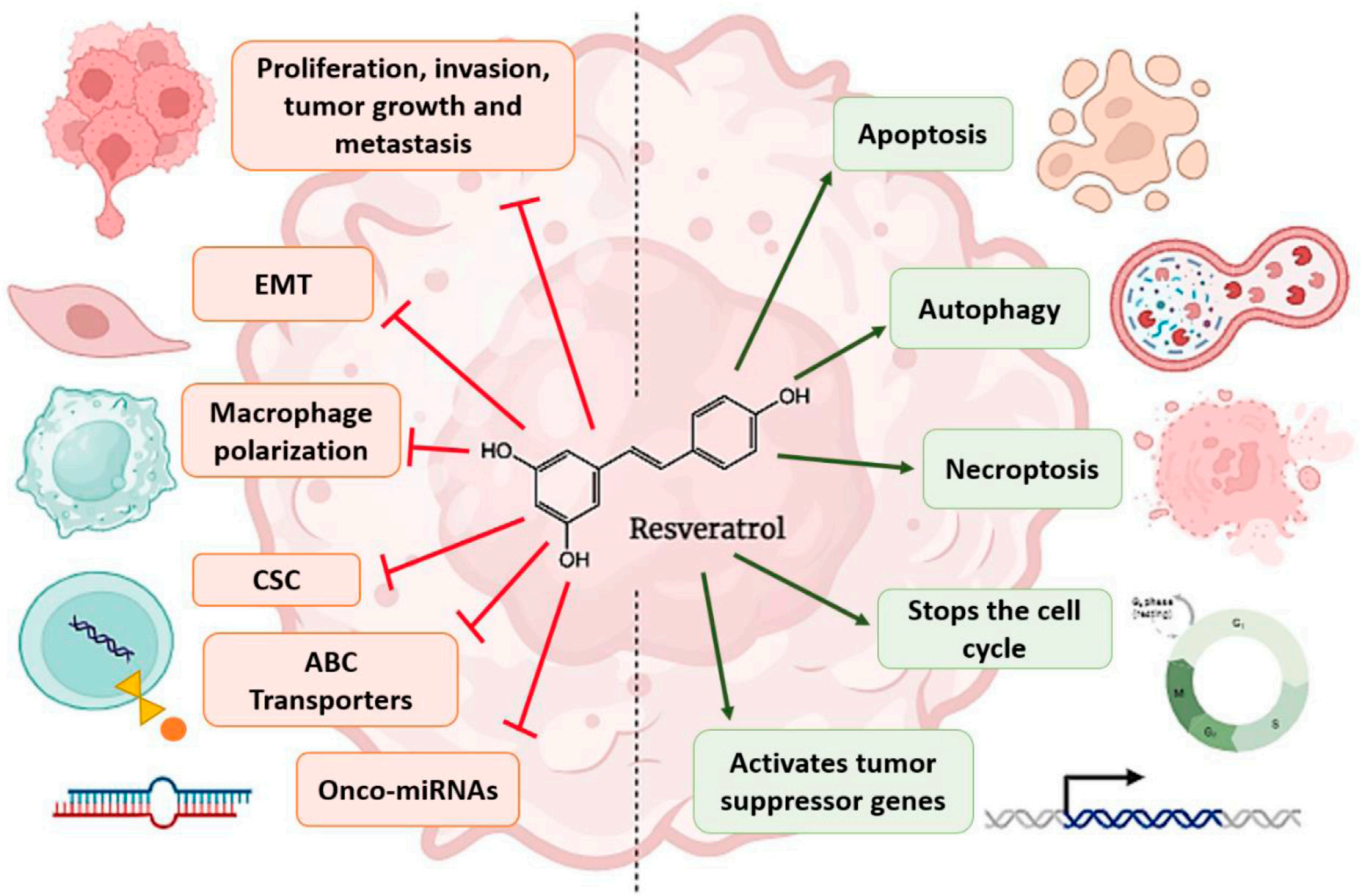
Factors regulated by RSV that lead to cancer chemo and radiosensitization. The arrows refer to promotion or increase, while the hammerhead lines refer to inhibition.

In the following figure, you can visualize in a more general way the events that RSV regulates to carry out the chemo and radiosensitization of cancer cells (Figure 4).

## 8 Conclusion

The acquisition of chemoresistance and radioresistance remains one of the main problems in the treatment of cancer patients. The fact that tumor cells develop multiple resistance mechanisms and that more than one mechanism can operate simultaneously complicates the success of anticancer treatments such as chemotherapy and radiotherapy.

In this review, we talk about the molecular mechanisms that lead to chemo- and radioresistance by cancer cells, and in addition, we try to concisely describe how RSV, in conjunction with treatments (radiotherapy and chemotherapy), manages to sensitize cancer cells, making them less resistant and favoring the effectiveness of the treatments. In addition, this article also summarizes the general effects of RSV treatment on cancer in an easy way to understand and shows the most recent studies that have addressed the issue of chemo- and radiosensitization of cancer cells by RSV. The RSV has the ability to modify and affect many molecular mechanisms that cause sensitization of cancer cells. Some of the most important mechanisms involved in sensitization we find transmembrane transport (decrease in drug transport proteins), regulation of the cell cycle (arrest of the cycle in the G1/S phases), decreased cell proliferation, activation of different types of cell death (apoptosis, necrosis, autophagy), inhibition of transcriptional factors such as NF-kB, blockage of DNA repair, reduction of inflammation due to the inhibition of COX-2, reduces the formation of CSCs by inhibiting oncogenic genes and onco-miRNAs, inhibition of epithelial-mesenchymal transition, activation of tumor suppressor genes, among others.

Based on everything analyzed and studied in this review, we conclude that RSV is undoubtedly an excellent candidate to be used as a complementary treatment to chemotherapy and radiotherapy since all the mechanisms and cellular targets that RSV regulates clearly favor the sensitization of cancer cells to these medications. This would improve the success rate of the treatments and undoubtedly improve the patients’ quality of life by reducing the treatment period.

## 9 Prospects

By looking at the signaling pathways and mechanisms that are affected or mediated by RSV in a tumor environment, we believe that RSV may be an ideal candidate as a complementary treatment to chemotherapy and radiotherapy. Although, there is a problem with the clinical use of RSV, and we are talking about its bioavailability. Many researchers have taken on the task of ending this barrier to use RSV more appropriately and efficiently. Today, many research works, such as those addressed in Sharifi-Rad’s article (Sharifi-Rad et al., 2021), show how targeted molecular therapy, specifically nanotechnology, shows to be a useful tool in improving the bioavailability of RSV.

Studies have even been carried out where co-encapsulation of RSV with other drugs, such as those used in chemotherapy, is proposed as in the works (Song et al., 2018; Zhang et al., 2022a) where they encapsulated RSV and DOX in nanoparticles of lactic-co-glycolic acid (PLGA) and hybrid lipid polymer (LPN) nanoparticles. This coencapsulation not only improved the half-life of DOX and RSV but also increased the concentrations of both molecules within the tumor, reducing the toxicity of DOX in healthy tissue and increasing the efficacy of DOX in overcoming the resistance of cancer cells (Cocetta et al., 2021). Therefore, these results suggest that nanoformulations protect and improve RSV’s stability and bioavailability, ensuring greater treatment efficiency. In addition, in conjunction with other drugs and therapies, it could even evade the resistance of cancer cells. However, further studies *in vivo* are needed to optimize nanocarrier and lipocarrier delivery systems.

Interestingly, new RSV analogues have also been reported to have increased bioavailability and have also been shown to possess anti-cancer properties (Ferraz da Costa et al., 2020). However, more studies are needed to understand its action and mechanisms of chemosensitization and radiosensitization.

### 9.1 Resource identification initiative

To take part in the Resource Identification Initiative, please use the corresponding catalog number and RRID in your current manuscript. For more information about the project and for steps on how to search for an RRID, please click here.

### 9.2 Life science identifiers

Life Science Identifiers (LSIDs) for ZOOBANK registered names or nomenclatural acts should be listed in the manuscript before the keywords with the following format:

urn:lsid:<Authority>:<Namespace>:<ObjectID>[:<Version>].
